# Phylogeographic Analysis of African Swine Fever Virus, Western Europe, 2018

**DOI:** 10.3201/eid2501.181535

**Published:** 2019-01

**Authors:** Mutien Garigliany, Daniel Desmecht, Marylène Tignon, Dominique Cassart, Christophe Lesenfant, Julien Paternostre, Rosario Volpe, Ann Brigitte Cay, Thierry van den Berg, Annick Linden

**Affiliations:** University of Liège, Sart Tilman, Belgium (M. Garigliany, D. Desmecht, D. Cassart, C. Lesenfant, J. Paternostre, R. Volpe, A. Linden);; Sciensano Animal Health, Brussels, Belgium (M. Tignon, A.B. Cay, T. van den Berg)

**Keywords:** African swine fever, Asfarviridae, wild boar, emergence, Belgium, viruses, Europe, African swine fever virus

## Abstract

In September 2018, African swine fever in wild boars was detected in Belgium. We used African swine fever–infected spleen samples to perform a phylogenetic analysis of the virus. The causative strain belongs to genotype II, and its closest relatives are viruses previously isolated in Ukraine, Belarus, Estonia, and European Russia.

African swine fever (ASF) is a devastating disease of domestic pigs and wild boars caused by a DNA arbovirus, African swine fever virus (ASFV), belonging to the family *Asfarviridae*. ASF is endemic in sub-Saharan Africa countries and has become more prevalent in the Caucasus region since its spread from eastern Africa to Georgia in 2007. The epizootic then spread to the surrounding countries, including the Russian Federation, and further to Belarus and Ukraine. In 2014, ASFV reached the European Union member states of Estonia, Latvia, Lithuania, and Poland; in 2016, Moldova; and, in 2017, the Czech Republic and Romania (*1*). 

On September 13, 2018, authorities in Belgium reported that ASF had been confirmed in 2 wild boars near the village of Étalle (49.6833° N, 5.6° E), in the province of Luxembourg, which is located 12 km from the border with France and 17 km from the country of Luxembourg. ASFV appears to have jumped a considerable distance from previously affected countries: ≈500 km from the border with the Czech Republic, 800 km from Hungary, and 1,200 km from the border with Romania. Since then, ≈75% of the wild boars found dead near the primordial spot have been found to be ASFV positive; a total of 96 positive results had been recorded as of October 16, 2018. 

To investigate the virus, we performed initial genetic characterization using standard genotyping procedure on virus DNA directly extracted from homogenized spleen or kidney tissues of each animal. First, we obtained a segment of the *B646L* gene by PCR as described by Gallardo et al. (*2*) and Ge et al. (*3*). The DNA sequence retrieved was identical in both animals (GenBank accession no. MH998358). We performed sequence alignments using ClustalW implemented in Geneious version 8.1.8 (https://www.geneious.com). We performed phylogenetic analysis using MEGA7 (http://www.megasoftware.net) and the Kimura 2-parameter substitution model, using the neighbor-joining method, as determined by a model selection analysis (Figure, panel A). The strain of ASFV found in Belgium clearly belonged to genotype II, which includes viruses that are circulating in both Eurasia and southern Africa.

**Figure:**
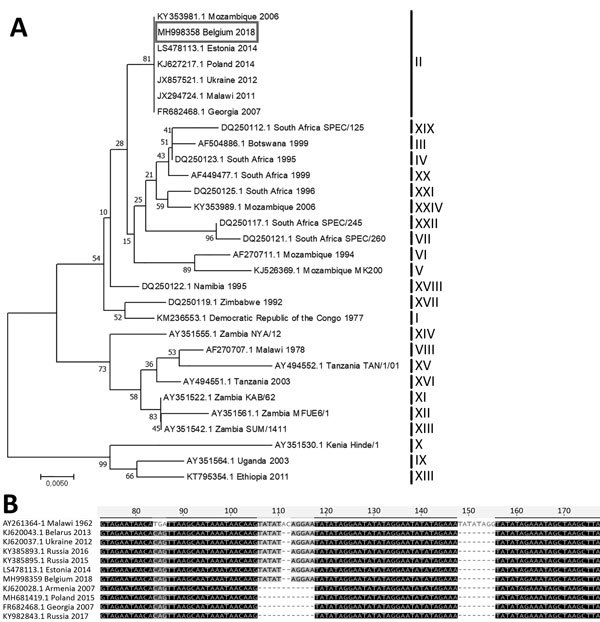
A) Evolutionary relationships of representative strains of African swine fever virus based on the neighbor-joining phylogeny of the partial p72 gene sequences. The phylogenetic analysis was performed using MEGA7 (http://www.megasoftware.net) and the Kimura 2-parameter substitution model, as determined by a model selection analysis. Bootstrap values (>70%, based on 500 replicates) for each node are given. GenBank accession numbers, country, and year of collection are indicated for each strain; for strains for which the year of collection is not known, the strain name is indicated. Corresponding genotypes are labeled I–XXIV. Box indicates the African swine fever sequence from Belgium generated during this study. Scale bar indicates nucleotide substitutions per site. B) Nucleotide sequence alignment of the partial intergenic region between the *I73R* and *I329L* genes from representative African swine fever virus strains. GenBank accession numbers, country, and year of collection are indicated.

To further define the most likely origin of this strain, we performed PCR targeting a ≈350-bp fragment in the variable intergenic region between the *I73R* and *I329L* genes, according to Gallardo et al. (*2*). Again, we retrieved identical DNA sequences from both animals (GenBank accession no. MH998359). Sequence alignments revealed that the isolate ASFV/Etalle/wb/2018 from Belgium contains a 10-nt (TATATAGGAA) insertion at position 106. It is therefore a so-called intergenic region (IGR) II variant, according to the nomenclature of Gallardo et al. (*2*). ASFV/Etalle/wb/2018 displays 100% identity with the sequences obtained from strains isolated in Ukraine in 2012, Belarus in 2013, Estonia in 2014, European Russia in 2015 and 2016, and China in 2018 (*3*,*4*), suggesting that the strain in Belgium most likely originates from one of these countries (Figure, panel B). Conversely, this insertion is absent in strains isolated in Armenia in 2007, in Georgia in 2007, in Poland in 2015, and in Siberia, Russia, in 2017 (*4*). Genotyping the p72 and IGR loci is compatible with the current state of the art for ASFV molecular epidemiology but still presents intrinsic limitations. A further genome-wide genotyping approach is expected to consolidate and bring more precision to the filiation revealed here.
